# Twinning of Pleomorphic Adenoma: A Case Report

**DOI:** 10.7759/cureus.6608

**Published:** 2020-01-09

**Authors:** Nishitha V Adiyodi, Joyce Sequeira, Anchal Mehra

**Affiliations:** 1 Oral and Maxillofacial Surgery, Yenepoya Dental College, Mangalore, IND

**Keywords:** pleomorphic adenoma, minor salivary gland tumor, upper lip

## Abstract

Pleomorphic adenoma is a common benign salivary gland tumor which presents as a painless swelling that gradually increases in size, if left untreated. It is often seen involving the parotid gland. However, pleomorphic adenoma has been reported to involve the minor salivary glands as well. In this report, we present two cases of pleomorphic adenoma originating from minor salivary glands of the upper lip, occurring in two male patients of the same age (44 years) with markedly similar history of duration, size, and site of the lesion. The tumor was excised in both patients and sent for histopathological analysis which showed features of pleomorphic adenoma confirming the diagnosis.

## Introduction

Pleomorphic adenoma is a common benign tumor of the salivary gland. These lesions account for two-thirds, or approximately 60%-65%, of all salivary gland tumors [1]. It shows a female predilection and is most commonly seen in the fourth to sixth decade of life. The tumor progresses as an asymptomatic slow growth over a prolonged period of time. The lesion originates most commonly from the major salivary glands, most commonly the parotid gland, although cases of occurrence in minor salivary glands have also been reported. Lips and palate are the most common sites; 20%-40% of all intraoral pleomorphic adenomas have been associated with the minor salivary gland. While the etiology of pleomorphic adenoma still remains elusive, it is known to be epithelial in origin, and clonal chromosome abnormalities with aberrations involving 8q12 and 12q15 have been implicated [2].

In this paper, we present two cases of pleomorphic adenomas involving the minor salivary glands, with strikingly similar clinical and demographic characteristics.

## Case presentation

Case 1

**Figure 1:**
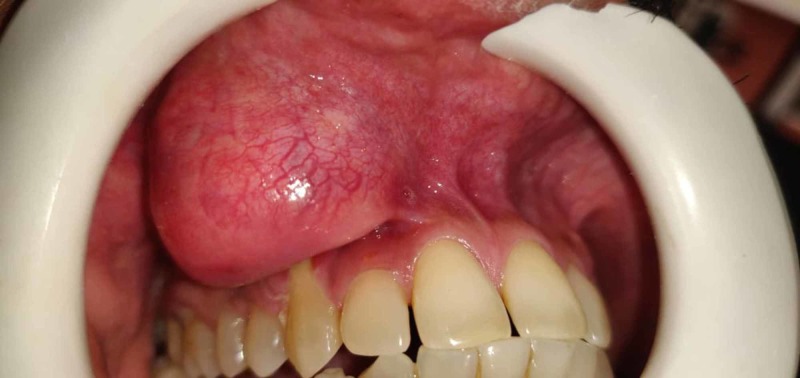
Preoperative view of the tumor in Case 1

A 44-year-old male patient visited the outpatient department (OPD) with a painless solitary growth intraorally in the upper right lip since five years. The growth initially appeared as a small lesion, which eventually grew to its present size. On examination, we found a well-defined, round, firm, non-tender, non-fluctuant, and mobile lesion with a size of approximately 3 × 3 cm on the right side of the upper lip. Class II gingival recession in relation to the right canine was noted. The overlying mucosa was pinkish in color, with evidence of superficial vascularity. Figure 1 shows the preoperative view of the tumor for the above-mentioned case. Intraoral periapical radiography of the pertinent region was obtained, which revealed no bone involvement. Differential diagnosis of the lesion included peripheral giant cell granuloma, minor salivary gland tumor, and lipoma for which surgical excision was planned.

Case 2

**Figure 2:**
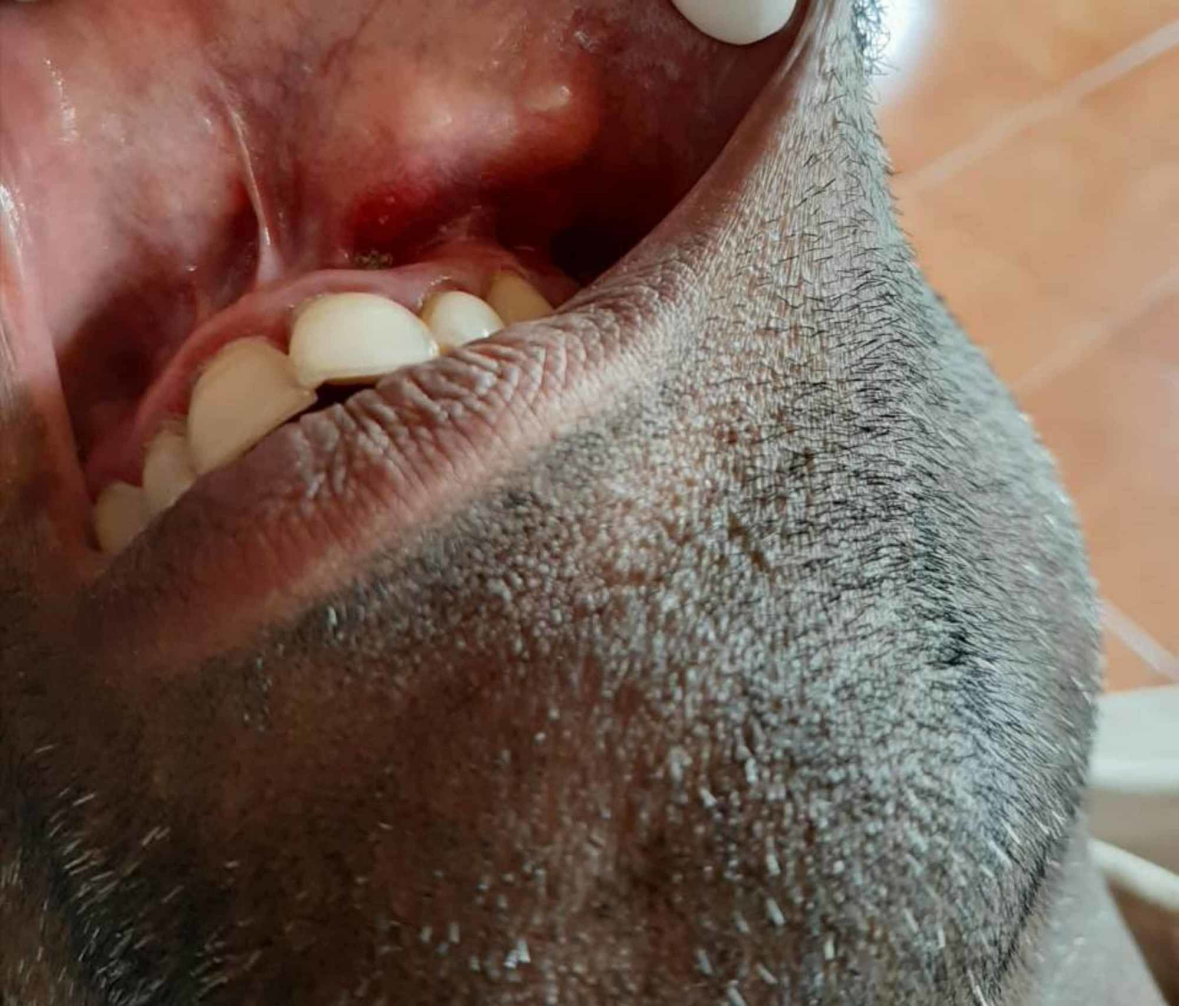
Preoperative view of the tumor in Case 2

A 44-year-old male patient reported to the OPD with a solitary swelling on the left side of the upper lip since five years, with intermittent pain in the region. The lesion started as a small growth, which subsequently increased to its current size. On examination, the mass was found to be a well-defined, round, firm, non-tender, non-fluctuant and mobile lesion of size approximately 4 × 3 cm on the left side of the upper lip. The overlying mucosa appeared normal, with superficial vascularity. Preoperative view of the tumor for Case 2 can be seen in Figure 2. Excision of the lesion was planned under the differential diagnosis of peripheral giant-cell granuloma, minor salivary gland tumor, and lipoma.

Surgical excision

**Figure 3:**
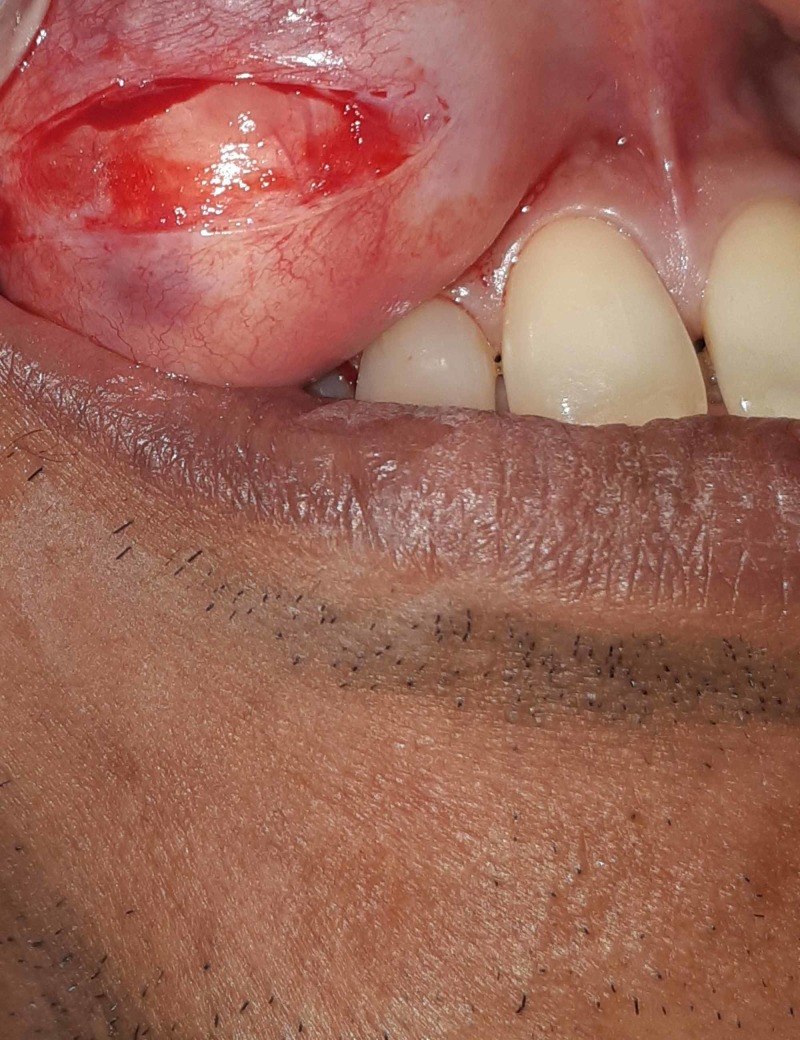
Surgical excision of the tumor in Case 1 Vestibular incision was given to excise the tumor.

**Figure 4:**
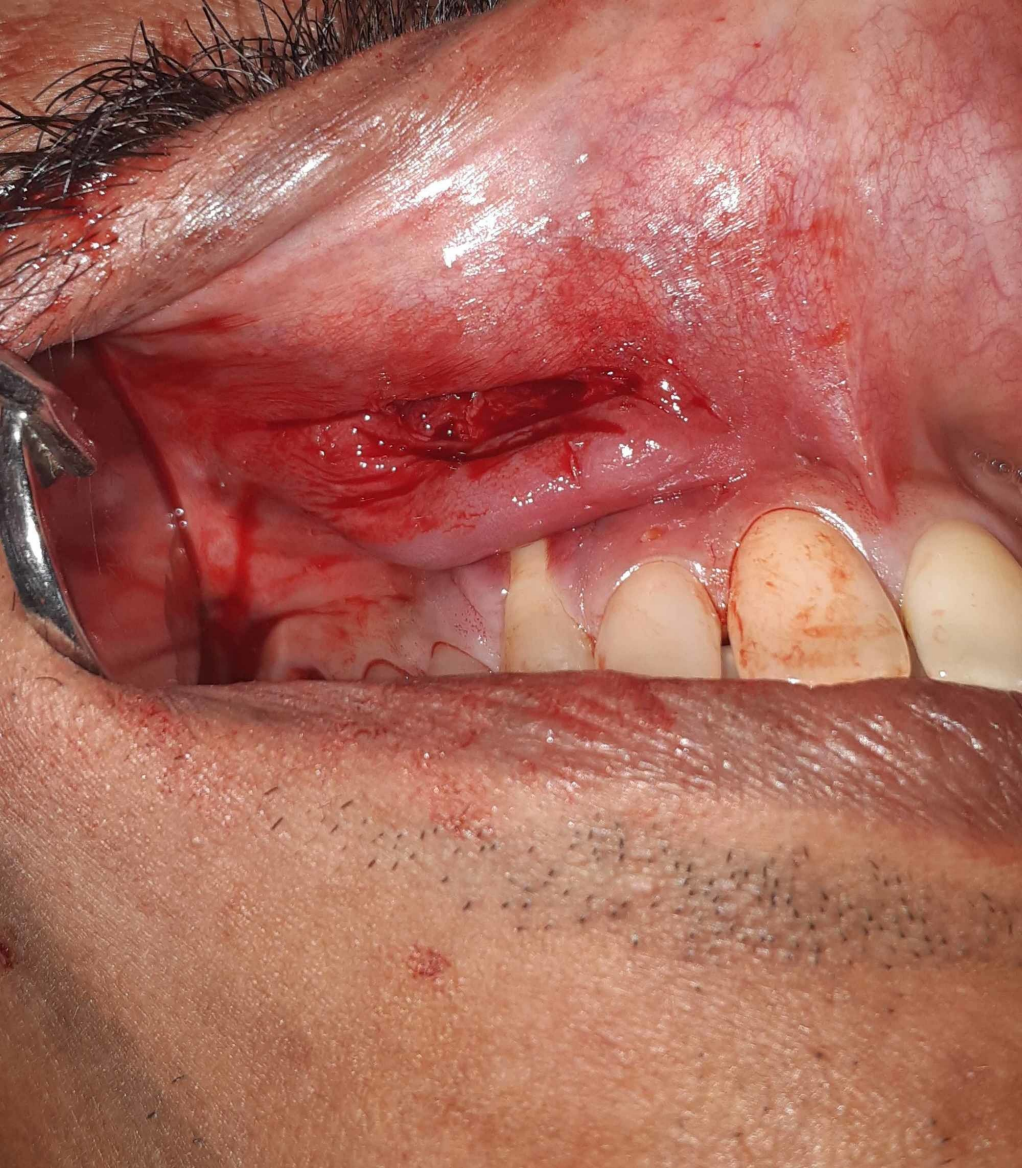
Intraoperative view of the area after the excision of the tumor in Case 1 Following submucosal dissection, the tumor was excised and any excess soft tissue present was removed before closure.

**Figure 5:**
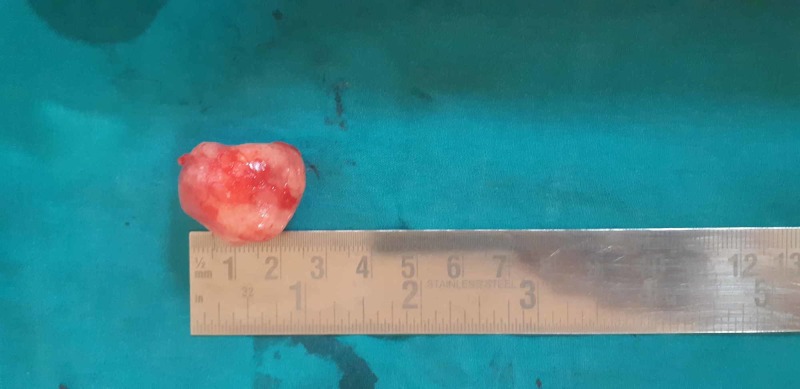
Excised tumor specimen of Case 1 Tumor of approximately 3 x 3 cm was excised and sent for histopathology.

**Figure 6:**
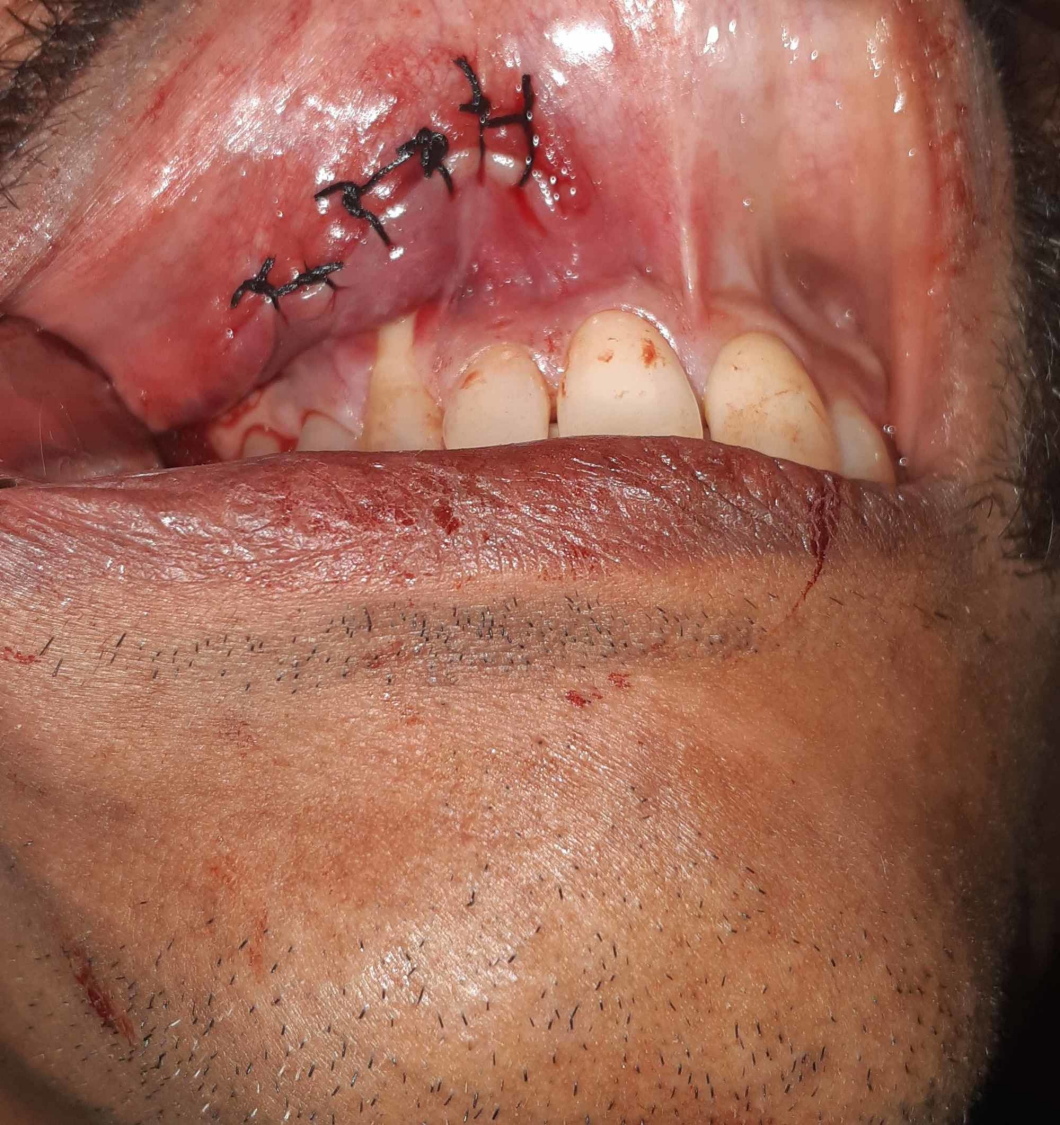
Closure with 3 - 0 silk suture of Case 1 Closure of the incision was done with simple interrupted sutures using 3 - 0 silk.

**Figure 7:**
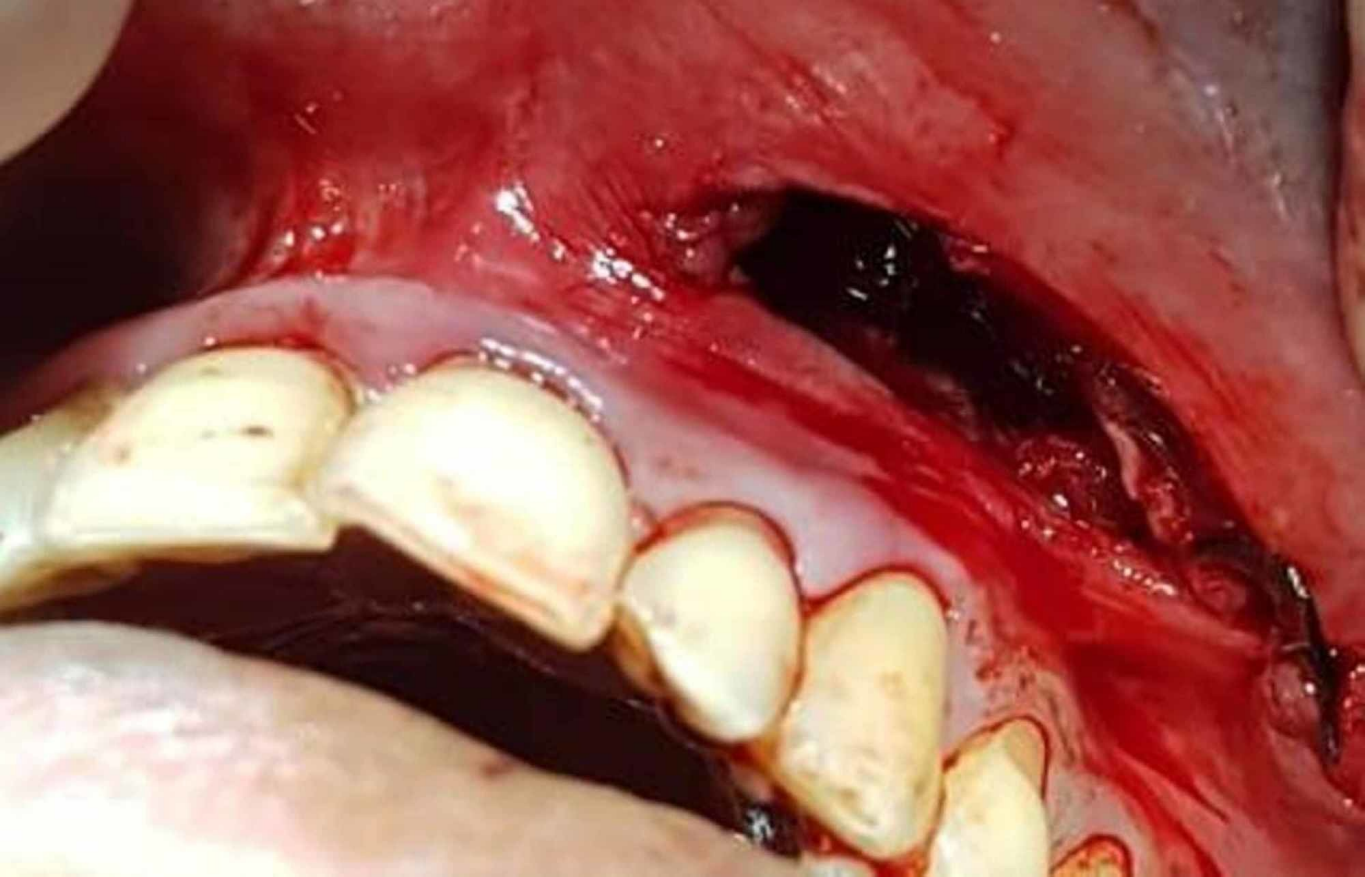
Intraoperative view of the site following surgical excision of the tumor in Case 2

**Figure 8:**
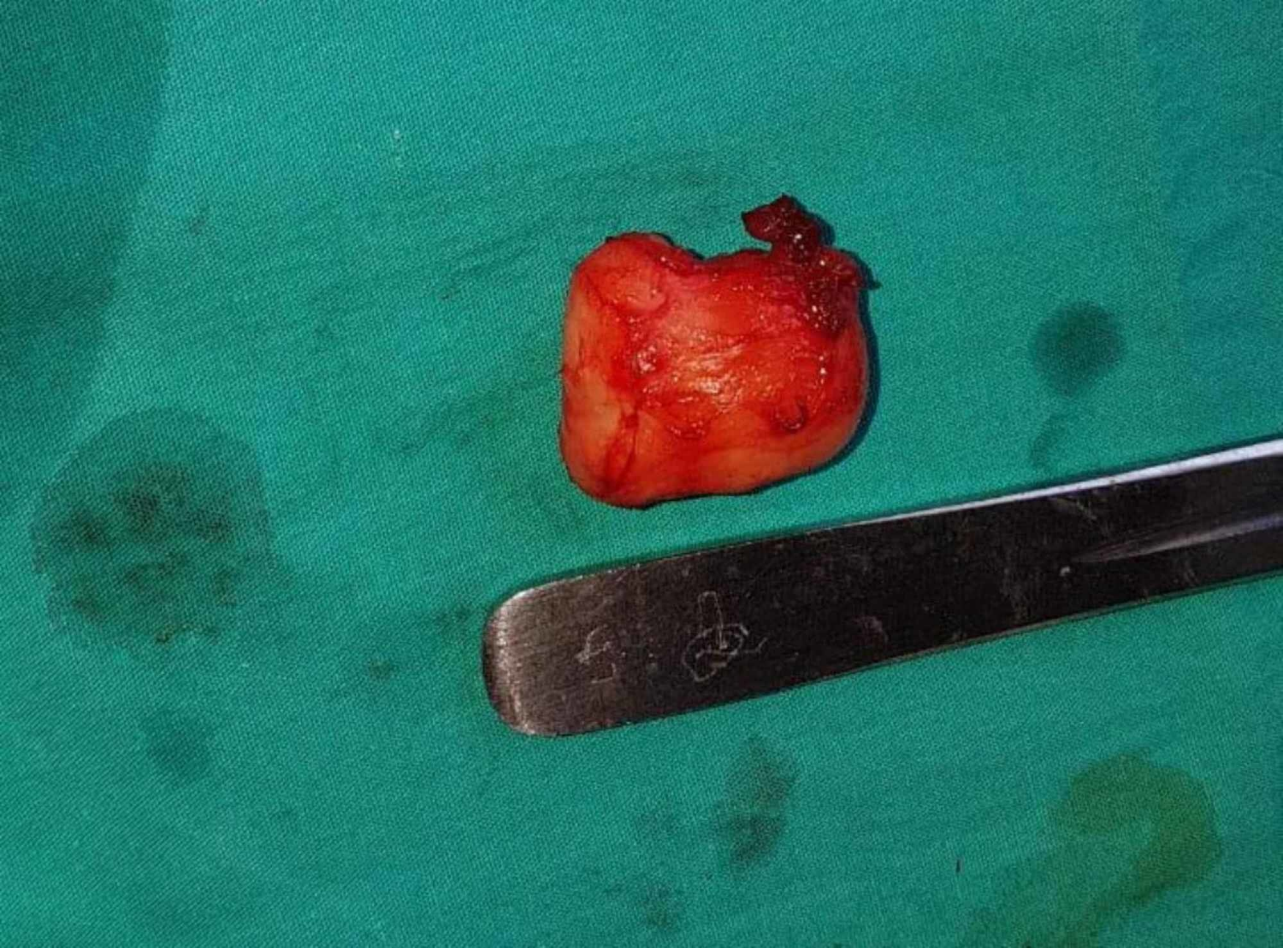
Excised tumor of approximately 4 x 4 cm in Case 2

**Figure 9:**
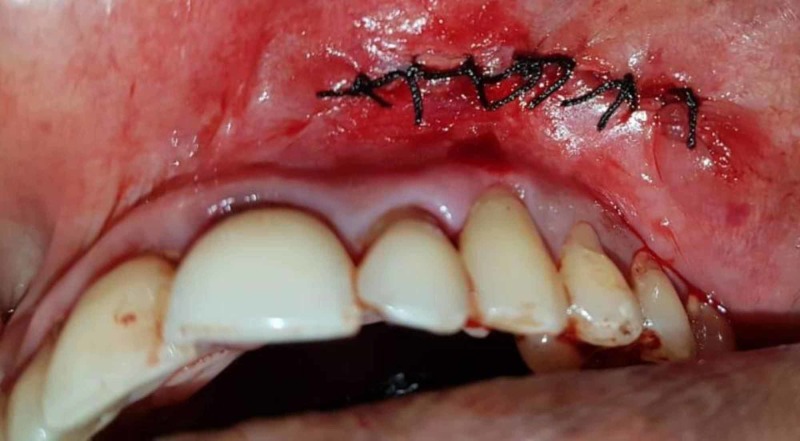
Closure done with 3 - 0 silk for Case 2 Closure of the surgical site done following the excision of the tumor with simple interrupted sutures using 3 - 0 silk.

**Figure 10:**
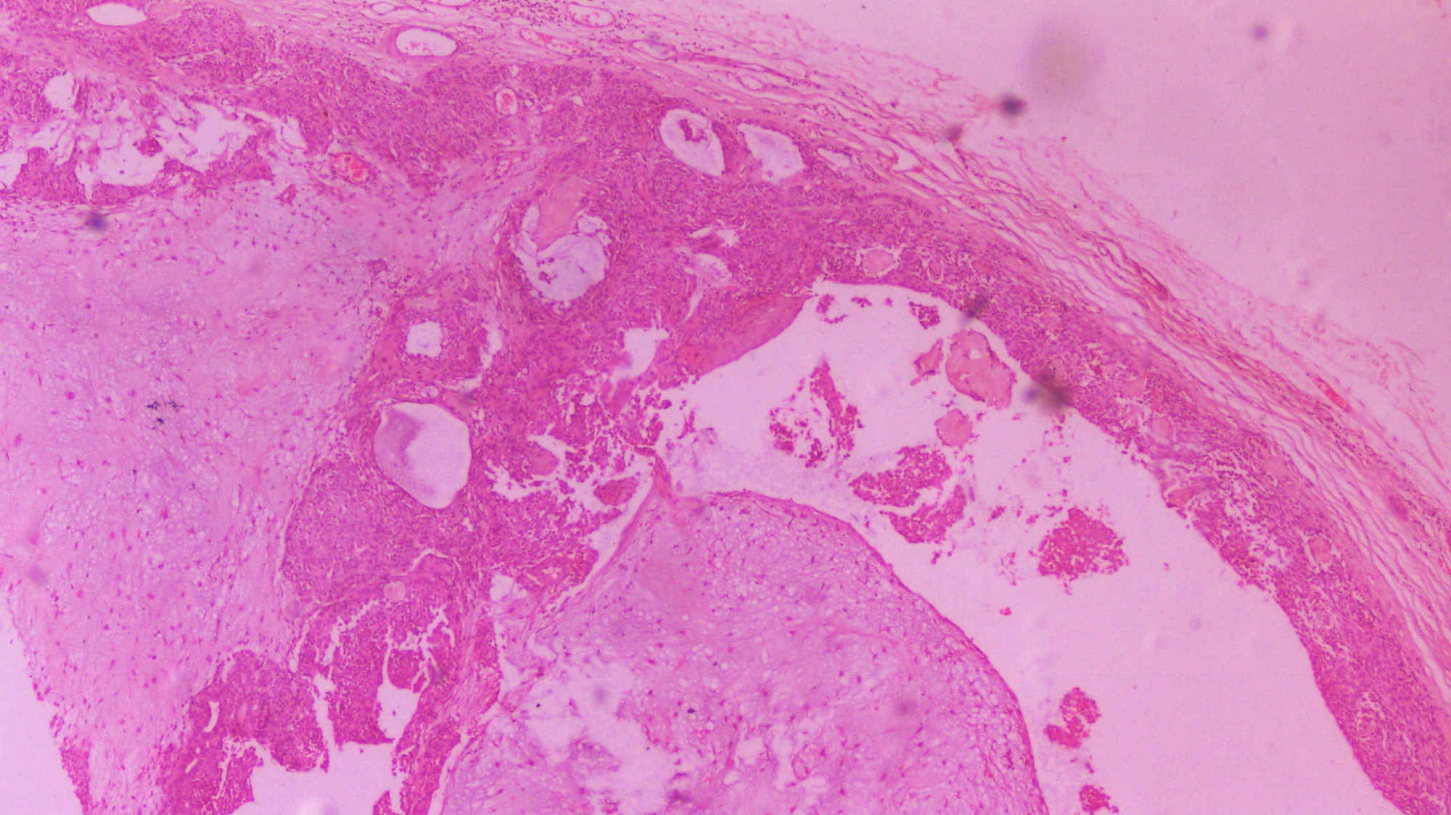
Histopathological section of the tumor Section shows well encapsulated lesion surrounded by fibrous capsule. The stroma consist of extensive zones of myxoid tissue with ovoid to spindle shaped myoepithelial cells and chondroid areas.

The same surgical procedure was used in both the cases. A vestibular incision was made over the lesion as seen in Figure 3. Submucosal dissection was then performed to expose the mass and release it from the underlying structures. The lesion was then removed from the maxillary vestibule as seen in Figures 4-5 (this can be seen in Figures 7-8 as well for Case 2). Extra soft tissue present in the vestibule was excised, followed by its closure with 3 - 0 silk as seen in Figures 6-9. The excised specimens were subjected to histopathology. The histopathology of both the cases revealed features of pleomorphic adenoma. The section showed a well-encapsulated lesion surrounded by a fibrous capsule. It also displayed numerous ductal and myoepithelial cells in the form of duct-like structures, strands and sheets, lined by cuboidal cells. The ductal spaces of variable dimensions complete with eosinophilic coagulum was noted. The stroma consisted of extensive zones of myxoid tissue with ovoid to spindle-shaped myoepithelial cells and chondroid areas. This can be seen in Figure 10.

## Discussion

Pleomorphic adenoma is a tumor that is often associated with major salivary gland especially the parotid gland. However, its involvement with minor salivary glands has been described as well. Shreshta et al. stated in their study that pleomorphic adenoma with respect to the upper lip is more common than those of the lower lip by a ratio of 6:1 [2]. Kroll and Hick reviewed 4042 cases of pleomorphic adenomas of the salivary glands and found that 445 originated in the minor salivary glands; 16.9% of the tumors originating in the minor salivary glands were located in the upper lip and 2.9% were in the lower lip [2]. The increased occurrence of tumors in the upper lip could be attributed to the fact that the upper lip develops from the fusion of three complex embryonic processes. Compared to the lower lip, the upper lip has a greater probability of embryonic cell nests being entrapped. This increases the risk of tumor formation in the upper lip [3]. Pleomorphic adenomas usually present as a well-circumcised mass. Tumors arising from minor salivary glands are histologically similar to those arising from the major salivary glands, although the former tend to be cellular with only a very minimal stromal and cartilaginous component [4]. Although an encapsulated, solid, mobile nodule, as seen in our cases, is characteristic of a benign lesion, a biopsy is necessary to confirm the absence of malignancy [5]. Minor salivary gland tumors may be seen clinically as a soft or firm mass, with most of them having a nodular, exophytic component. The invasiveness of the tumor cannot be confirmed if the ulceration of the nodular mass is seen. Tumors that contain large cystic cavities and abundant mucin may be clinically soft on palpation [5]. The patient may not be aware of their existence as these lesions are clinically asymptomatic and it may be discovered on routine examination.

Pleomorphic adenoma is essentially a benign tumor. Nevertheless, aggressive behavior of the lesion has been described; there have been instances of the tumor invading neighboring vessels in the absence of any other features of malignancy [6]. In their case series, Yih et al. observed that pleomorphic adenoma is the most common minor salivary gland tumor, followed by mucoepidermoid carcinoma; they found that among the minor salivary gland tumors occurring in the lips, benign tumors are usually seen in the upper lip; whereas, malignant tumors are usually seen in the lower lip [7-8]. Jansisyanont et al. [9] also stated that pleomorphic adenoma was the most common benign tumor involving the minor salivary gland. Vicente et al. [10] observed an increased risk of recurrence with incomplete excision of the minor salivary gland. Furthermore, the possibility of malignant transformation of the tumor should be taken into consideration, and periodic clinical evaluation of the patients is necessary. There was one particular study by Martinell et al. [11] that showed the association of simian virus with the occurrence of pleomorphic adenoma of the parotid glands. No other predisposing or risk factors have been attributed to the occurrence of pleomorphic adenoma in literature. 

## Conclusions

Pleomorphic adenoma is a benign neoplasm most commonly seen originating from the parotid gland. Here, we reported two unusual situations of pleomorphic adenoma involving the minor salivary glands with identical clinical and demographic characteristics with respect to location, size, and history of the lesion. Such an occurrence at the same period of time is quite rare and noteworthy.
